# Plant diversity and identity effects on predatory nematodes and their prey

**DOI:** 10.1002/ece3.1337

**Published:** 2015-01-23

**Authors:** Olga Kostenko, Henk Duyts, Saskia Grootemaat, Gerlinde B De Deyn, T Martijn Bezemer

**Affiliations:** 1 Department of Terrestrial Ecology, Netherlands Institute of Ecology (NIOO-KNAW)PO Box 50, Wageningen, 6700 AB, The Netherlands; 2 Department of Soil Quality, Wageningen UniversityP.O. Box 47, Wageningen, 6700 AA, The Netherlands

**Keywords:** Belowground, biodiversity, natural enemies, plant diversity, predation, soil biota

## Abstract

There is considerable evidence that both plant diversity and plant identity can influence the level of predation and predator abundance aboveground. However, how the level of predation in the soil and the abundance of predatory soil fauna are related to plant diversity and identity remains largely unknown. In a biodiversity field experiment, we examined the effects of plant diversity and identity on the infectivity of entomopathogenic nematodes (EPNs, *Heterorhabditis* and *Steinernema* spp.), which prey on soil arthropods, and abundance of carnivorous non-EPNs, which are predators of other nematode groups. To obtain a comprehensive view of the potential prey/food availability, we also quantified the abundance of soil insects and nonpredatory nematodes and the root biomass in the experimental plots. We used structural equation modeling (SEM) to investigate possible pathways by which plant diversity and identity may affect EPN infectivity and the abundance of carnivorous non-EPNs. *Heterorhabditis* spp. infectivity and the abundance of carnivorous non-EPNs were not directly related to plant diversity or the proportion of legumes, grasses and forbs in the plant community. However, *Steinernema* spp. infectivity was higher in monocultures of *Festuca rubra* and *Trifolium pratense* than in monocultures of the other six plant species. SEM revealed that legumes positively affected *Steinernema* infectivity, whereas plant diversity indirectly affected the infectivity of *Heterorhabditis*EPNs via effects on the abundance of soil insects. The abundance of prey (soil insects and root-feeding, bacterivorous, and fungivorous nematodes) increased with higher plant diversity. The abundance of prey nematodes was also positively affected by legumes. These plant community effects could not be explained by changes in root biomass. Our results show that plant diversity and identity effects on belowground biota (particularly soil nematode community) can differ between organisms that belong to the same feeding guild and that generalizations about plant diversity effects on soil organisms should be made with great caution.

## Introduction

Biodiversity is rapidly declining worldwide, and many studies have shown that this can result in significant negative effects on ecosystem processes, including economically important ecosystem services such as control of pest insects (Cardinale et al. 2003; Brussaard 2012). Most studies investigating the effects of species loss on ecosystem services and processes have focused on the aboveground effects of plant species richness hereafter named “plant diversity” and show that a decline in plant diversity negatively affects the abundance or diversity of predators and parasitoids of foliar feeding herbivores (Thies and Tscharntke 1999; Haddad et al. 2009; Scherber et al. 2010). However, how the level of predation in the soil and the abundance of predatory soil organisms are related to the diversity and identity of the plant community is less well understood, and the few studies addressing this question have focused on carabid assemblages, predatory nematodes, and predatory macrofauna (Wardle et al. 2003; Harvey et al. 2008; Scherber et al. 2010).

Entomopathogenic nematodes (EPNs) of the genera *Steinernema* and *Heterorhabditis* (Rhabditida: Steinernematidae and Heterorhabditidae) are natural enemies of insects or other arthropods that live in the soil or close to the soil surface (Kaya and Gaugler 1993). EPNs are present in the soil of most terrestrial ecosystems and used in pest management programs worldwide. They spend part of their life cycle in soil as free-living nonfeeding infective juveniles and the other part in insect bodies which they infect and kill. EPNs are sensitive to abiotic factors, such as temperature and moisture, and biotic factors such as competition and natural enemies (e.g., nematophagous fungi, Collembolans and mites) (Lewis et al. 2006). Studies that have estimated the effects of intercropping on the presence and infectivity of EPNs show that heterogeneous vegetation in agricultural systems can serve as a refuge for EPNs (Lawrence et al. 2006; Jabbour and Barbercheck 2008). How the infectivity and natural occurrence of EPNs are related to the diversity or composition of natural plant communities is less well known.

Carnivorous non-EP nematodes feed predominantly on other nematodes and have evolved special features for ingesting nematode prey, such as root-feeding, bacterivorous, fungivorous and omnivorous nematodes (Yeates et al. 1993). Previous studies on the effects of plant diversity on non-EPNs mainly focused on functional shifts in nematode composition and have reported weak or nonexisting effects of plant diversity on carnivorous non-EPNs (e.g., De Deyn et al. 2004a; Viketoft et al. 2009; Eisenhauer et al. 2011). However, the mechanisms of these weak responses have remained largely unclear.

Root-feeding insects and nematodes use plant roots as a food source and can be directly affected by changes in root diversity or biomass production (De Deyn et al. 2004b). Increases in root biomass can also indirectly enhance the abundance of organisms that are part of the decomposer subsystem of the soil food web, such as bacterivorous and fungivorous nematodes, via increased amounts of litter or root exudates that serve as the basal resource for decomposition (Wardle et al. 2003). According to the diversity-trophic structure hypothesis (Hutchinson 1959), such increases in the abundance of soil organisms that inhabit lower trophic levels may then positively affect predatory soil organisms, as their prey density increases. Alternatively, increases in plant diversity and biomass production may affect the abundance of soil predatory organisms directly, for example, by providing habitat or refuge in the case of abiotic extremes or competition (Lawrence et al. 2006). Therefore, the relationship between plant diversity, biomass, and higher trophic levels comprises a complex network of direct and indirect links, and it is not known how the interactions in these multitrophic networks operate. Here, we use structural equation modeling (SEM) to examine plant diversity effects on belowground multitrophic networks with a particular focus on EPNs and other carnivorous nematodes. SEM is a multivariate method that can be used to examine how alternative pathways in networks with direct and indirect relationships may contribute to the observed species responses to experimental treatments (Grace 2006).

Several studies have argued that the effects of plant diversity on other organisms are not directly due to the number of plant species per se, but rather due to the abundance of certain plant species or functional groups in the plant community (e.g., Spehn et al. 2000; Gastine et al. 2003; Wardle et al. 2003; De Deyn et al. 2004a; Viketoft et al. 2009). For example, densities of aboveground invertebrates, including predatory arthropods, are often higher in plant communities that contain leguminous species, most likely because the nutritional quality of plant tissues is often higher in communities that contain nitrogen-fixing plant species (e.g., Koricheva et al. 2000; Haddad et al. 2009). Many studies that have examined effects of plant identity on the abundance of carnivorous nematodes in grasslands did not find significant effects (Wardle et al. 2003; De Deyn et al. 2004a; Viketoft et al. 2009). However, plant diversity effects can be mediated by the changes in the abundance of the lower trophic level nematodes, and hence, the abundances of non-EP carnivorous nematodes and infectivity of EPNs in relation to the diversity and composition of the plant community warrant the inclusion of prey abundance.

In this study, we use a grassland biodiversity experiment, in which the diversity of the plant communities was manipulated and maintained, to examine the effects of plant diversity and identity on the infectivity of EPNs and abundance of carnivorous non-EPNs. To estimate the potential prey or food availability for EPNs and carnivorous non-EPNs, we also determined root biomass, the number of root-feeding, fungivorous, bacterivorous, and omnivorous nematodes, and root-feeding insects in soil samples. We hypothesized that (1) increased plant diversity will enhance EPN infectivity, the abundance of carnivorous non-EPN and prey nematodes, abundance of soil insects, and root biomass and that (2) plant functional groups and (3) plant species in monocultures will strongly differ in their effect on the densities of belowground organisms. In particular, we predict that the abundances of soil organisms will be positively related to the cover of legumes in the plant community. Finally, we examined whether the relationship between plant diversity, identity, and predation in the soil could be explained by changes in root biomass and/or prey abundances.

## Materials and methods

### Field site

A detailed description of the design of the field experiment has been presented elsewhere (Kostenko et al. 2012). In brief, in 2008, 70 experimental plots of 3 × 3 m separated by 1-m-wide lanes were setup in a nature restoration grassland area that had been agricultural land until 1996 (de Mossel, Ede, the Netherlands). The experimental area was fenced to exclude large vertebrate herbivores. The plots were sown with 1, 2, 4, or 9 plant species drawn from a pool of 12 grassland species including three grasses (*Anthoxanthum odoratum* L., *Agrostis capillaris* L., and *Festuca rubra* L.), three legumes (*Lotus corniculatus* L., *Trifolium arvense* L., and *Trifolium repens* L.), and six forbs (*Achillea millefolium* L., *Hypochaeris radicata* L., *Leucanthemum vulgare* Lamk., *Tanacetum vulgare* L., *Tripleurospermum maritimum* L. W. D. J. Koch, and *Plantago lanceolata* L.). Each diversity level was replicated with several different mixtures in order to avoid confounding effects of species identity and diversity. Each of the sown plant species mixtures and monocultures was replicated twice using a complete randomized design. At the moment of sampling, there were 16 monocultures; 18 plots with two species, 22 plots with four, six plots with nine species, and four plots were kept free of all vegetation and served as “bare soil” treatment. Four remaining plots were excluded from the experiment due to poor establishment. There were no monocultures of *A. odoratum*, *A. capillaris*, *T. arvense,* and *T. maritimum*, but these species were present in the mixtures. Experimental plots were not mown, but hand-weeded during the growing season in 2009 and 2010 (from the end April until end August) to maintain the sown species composition. All soil samples were collected in September 2010.

### Infection bioassay

To assess the EPN infectivity in the experimental plots, we used a standard laboratory *Galleria*-bait method (Bedding and Akhurst 1975). Soil for the assay was collected from each experimental plot by taking 25 soil cores of 15 cm depth and 5 cm diameter from the inner 2.5 × 2.5 m square in a regular 0.5 × 0.5 m grid. The samples were pooled per plot. Plastic containers (10 × 10 × 5 cm) were filled with 250 g soil from each plot. The soil was adjusted to field capacity (15%) by adding de-mineralized water. There were four containers per plot. Into each container, four final instar *G. mellonella* larvae were placed on the soil surface; the containers were closed and flipped over so that the larvae were covered by soil. The insect larvae were obtained from Kreca V. O. F. (Ermelo, the Netherlands). The containers were kept in a dark climate chamber under controlled conditions at 22°C, 50–60% humidity. After 1 week, all the larvae were retrieved from the soil and incubated individually in the labeled plastic vials (3 cm diameter, 5 cm height) in the climate chamber. Seven days later, all larvae were dissected and examined using a stereo microscope in order to assess infection by *Heterorhabditis* or *Steinernema* EPNs. Assessments were based on the color of the cadaver and the morphology of adult nematodes found in the dissected larvae (Stock and Hunt 2005). Because EPNs typically kill their hosts within 48 h (Kaya and Gaugler 1993), the 2 weeks scoring period virtually assured that we observed all nematode-imposed mortality. All EPN-infected larvae were dead before the dissection. We also recorded whether larvae died from fungal or bacterial infection. We grouped these larvae together as larvae that died from other causes.

### Soil nematode extraction and identification

The soil for assessing the nematode community size and composition was a 100-ml subsample from the pooled soil collected for EPN infectivity bioassay. Soil moisture content was determined on another soil subsample of each plot by drying 50 g of fresh soil for 3 days at 120°C. Nematodes were extracted from 100 mL fresh soil using Oostenbrink elutriators (Oostenbrink 1960; see Appendix S1 for details). Nematode densities were calculated per 100 g dry weight soil. Nematodes were categorized into feeding guilds according to Yeates et al. (1993), Andrássy (2005) and personal communication with a specialist in nematode taxonomy and biology (Prof Tom Bongers; Table S1, Appendix S1). We considered nematodes as being carnivorous if there is evidence in literature that they consume other nematodes, although some of the listed carnivores might also feed on other organisms, for example, bacteria (see Table S1 for details).

### Root biomass

To determine community standing root biomass, three soil cores of 10 cm depth and 2.5 cm diameter were taken 1 m apart along a diagonal transect within each plot that started 50 cm from the edge of the plot. In the laboratory, the weight of the soil in each core was determined, and all root material was washed, oven-dried at 70°C, and weighed. Total root biomass was calculated as root dry weight per 100 g dry soil.

### Soil insects

To estimate the abundance of soil-dwelling insects, four soil cores of 12.5 cm diameter and 15 cm deep were collected from four randomly selected locations within the inner 2.5 × 2.5 m square of each plot. In the laboratory, each soil sample was weighed and then hand-sorted. All visible arthropods were collected and stored in 70% ethanol in labeled Eppendorf tubes. The arthropods were categorized as white grub larvae (scarab beetle larvae), wireworms (*Elateridae* beetle larvae), other insect larvae (*Lepidoptera*, *Diptera*, and other *Coleoptera*), and adult beetles (*Coleoptera*). The abundance of soil insects was expressed per 100 g dry weight soil.

### Statistical analyses

All univariate analyses were performed using R statistical language, version 2.15.1 (R Development Core Team 2012). Percentage data were arcsine square-root-transformed; biomass and prey nematode data were log-transformed; insect and carnivorous nematodes data were square-root-transformed to meet the requirements of normality and homoscedasticity of errors. If the assumptions were still violated, nonparametric tests were used to analyze the data (for these analyses chi-square is reported). Because there were four containers per plot, the effects of plant diversity, monoculture identity, and proportion of legumes, grasses, or forbs in the vegetation on %EPN infectivity were analyzed using linear mixed models with plot identity as random factor. General linear models were used to test the effects of plant diversity, monoculture identity, and proportion of legumes, grasses, or other forbs in the vegetation on nematode and insect abundances, root biomass, and soil moisture content. Plant diversity was included as continuous variable to test for linear effects. We also repeated the analyses by excluding the bare plots. Individual comparisons between monocultures were based on a Tukey's HSD test. Due to the low number of insects recovered from monocultures, the effects of monoculture identity on the soil insect abundance were not tested. To determine whether there was a relationship between prey nematode community composition and plant diversity, we used multivariate principal component analysis (PCA) and redundancy analysis (RDA) in CANOCO version 5.03 (Šmilauer and Lepš 2014).

### Structural equation modeling

**Figure 1:**
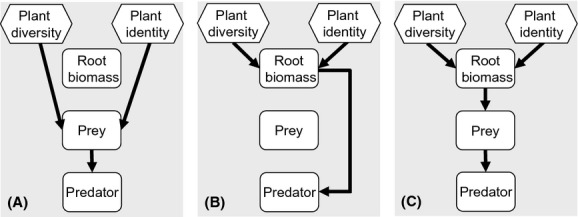
Three alternative hypothetical pathways between plant diversity and identity, root biomass, and prey and predator abundances that were tested by structural equation modeling. The hypothetical pathways A, B, and C are explained in the text.

We tested three alternative pathways linking plant diversity and identity to EPN infectivity or predatory nematode abundance via changes in prey abundance (A, Fig.1); via changes in root biomass (B, Fig.1); and via changes in root biomass that subsequently controls prey abundance (C, Fig.1). Separate models were developed for *Heterorhabditis* infectivity, *Steinernema* infectivity, and carnivorous non-EPN abundance. For EPN models, we included soil insects as prey, and for non-EPNs model, we included the total of root-feeding, bacterivorous and fungivorous nematodes as prey. Omnivorous nematodes were not included in the model as they also can feed on other food sources, such as bacteria or fungi. All plots were used in the analysis, and data were transformed in the same way as for univariate analysis. The likelihood ratios and chi-squared tests were used to determine whether the model-implied variance–covariance matrix differed from the observed variance–covariance matrix and to perform model simplification. The nonsignificant terms were removed from the initial model, and the model that best fitted our data was selected. This model was used to determine which of the proposed hypotheses best explained the relationship between plant diversity and identity and EPN infectivity or carnivorous non-EPN abundance (see Appendix S2 for more details). SEM was performed using sem package for R.

## Results

### Predator responses

**Figure 2:**
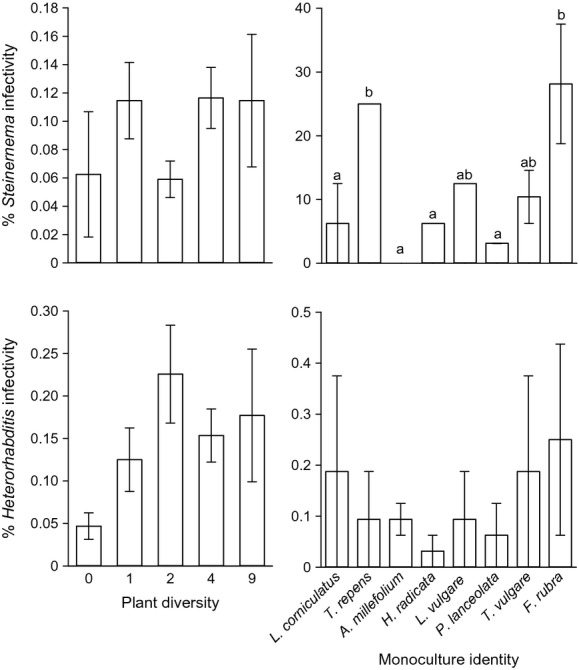
Effect of plant diversity and monoculture identity on mortality of *G. mellonella* larvae caused by *Steinernema* and *Heterorhabditis* spp. Means are calculated based on average values per plot ± between plot SE (if SE is not displayed, the mortality of *G. mellonella* larvae was equal in both plots). Different letters denote significant differences between monocultures (*P* < 0.05) based on linear mixed model with plot identity as random factor.

Average total mortality of *Galleria* larvae in the bioassay was 78%, of which 21% were infected by *Heterorhabditis* and 12% by *Steinernema,* while the other 43% died of other causes. Neither plant diversity nor the proportion of plant functional groups in the mixtures significantly affected infectivity by *Heterorhabditis* spp. (Table1). However, the *Heterorhabditis* infectivity was on average three times lower in the bare compare to vegetated plots (0.11 ± 0.03% and 0.27 ± 0.03%, respectively, Fig.2). Infectivity of *Heterorhabditis* spp. did not differ among monocultures (*F*_7,8_ = 0.31, *P* = 0.93). There was no significant effect of plant diversity on the infectivity of *Steinernema* spp. (Table1). However, the *Steinernema* infectivity was lower in plots where forbs were abundant; this effect was significant only when bare plots were excluded from the analysis (Table1). The infectivity by *Steinernema* spp. varied significantly among monocultures (*F*_7,8_ = 3.67, *P* = 0.044; Fig.2) and was highest in the monocultures of *F. rubra* and *T. repens*. The percentage of the larvae that died due to other causes was not affected by plant diversity or by the plant functional groups (Table1) and did not differ among monocultures (*F*_7,8_ = 1.27, *P* = 0.37).

**Table 1:** Effects of plant diversity, proportion of legumes, grasses, and forbs on the infectivity of entomopathogens, abundance of other nematodes, soil insect abundance, and community root biomass

	Plant diversity	Legumes	Grasses	Forbs
Bare plots included				
*Predator responses*				
*Heterorhabditis* infectivity	1.15	0.003	1.85	0.09
*Steinernema* infectivity	1.25	3.26	1.32	3.27
Other mortality	0.46	0.22	0.19	0.013
Carnivorous nematodes	0.08	0.44	0.24	2.56
*Prey responses*				
Root-feeding nematodes	↑5.95*	↑16.18***	↑5.81*	9.56**↓
Bacterivorous nematodes	↑8.68**	↑9.30**	1.90	0.001
Fungivorous nematodes	↑7.81**	↑9.34**	4.59*↓	2.38
Omnivorous nematodes	↑5.81*	1.88	0.95	2.32
Insect abundance	↑5.83*	0.67	1.73	0.14
*Community root biomass*	1.74	0.0004	↑4.51*	0.0003
Bare plots not included				
*Predator responses*				
*Heterorhabditis* infectivity	0.16	0.051	1.32	0.58
*Steinernema* infectivity	0.60	2.90	1.08	4.88*↓
Other mortality	0.01	0.10	0.08	0.23
Carnivorous nematodes	1.32	0.71	0.46	1.59
*Prey responses*				
Root-feeding nematodes	0.69	↑13.34***	↑4.00*	20.18***↓
Bacterivorous nematodes	↑(3.84)*	↑7.58**	2.97	0.64
Fungivorous nematodes	0.52	↑6.16*	↑7.06*	0.06
Omnivorous nematodes	0.46	0.88	2.19	0.19
Insect abundance	↑(3.62)*	0.98	1.21	0.01
*Community root biomass*	0.22	0.19	2.81	1.13

*F*-values are shown of linear mixed models for infectivity of EPNs and other mortality causes and general linear models for other response variables. The respective explanatory variable in those models was fitted first. Asterisks indicate significant effect at

*** *P* < 0.001;

** *P* < 0.01; and

* *P* < 0.05; the brackets indicate marginally significant effect at *P* < 0.06; the absence of asterisks indicates no significant effect. ↑ indicates positive effect, and ↓ indicates negative effect.

**Figure 3:**
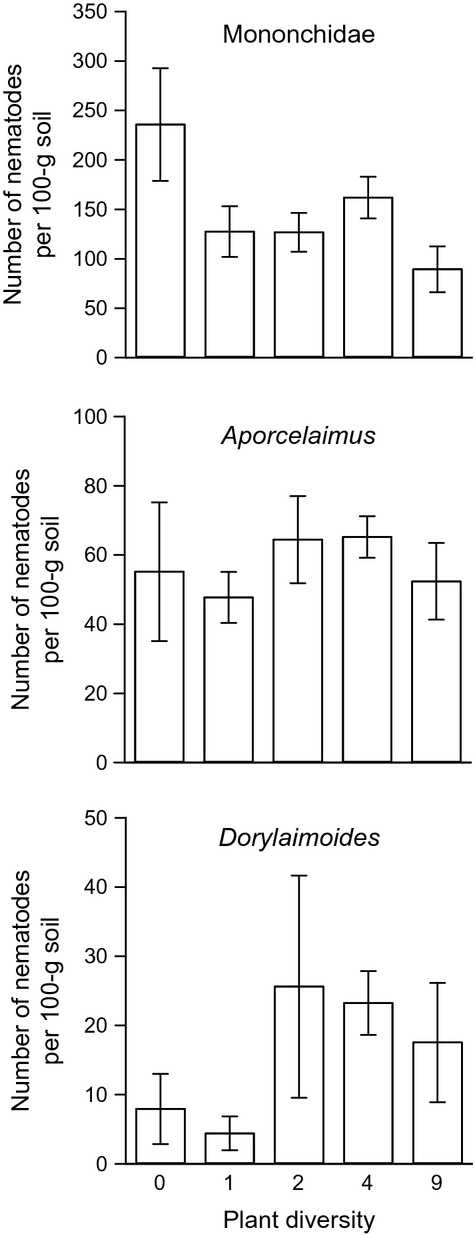
Effect of plant diversity on the abundance of carnivorous nematodes of family *Mononchidae* and genera *Aporcelaimus* and *Dorylaimoides*. The number of nematodes was calculated per 100 g dry weight soil.

The abundance of carnivorous non-EPNs was not significantly affected by plant diversity or by the plant functional groups (Table1) and did not differ among monocultures (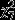 = 3.09, *P* = 0.88). Nematodes of the family *Mononchidae* and of the genera *Aporcelaimus* and *Dorylaimoides* were the most dominant carnivorous non-EPNs in our study (Table S1). The abundance of *Mononchidae* was highest in bare plots (236 ± 57 nematodes per 100 g soil) and lowest in nine species plots (89 ± 23 nematodes per 100 g soil); however, there was no significant effect of plant diversity on the *Mononchidae* abundance (*F*_1,64_ = 0.39, *P* = 0.53, Fig.3). The abundance of *Aporcelaimus* was not affected by increase in plant diversity (*F*_1,64_ = 1.02, *P* = 0.32, Fig.3), whereas *Dorylaimoides* nematode abundance increased with increasing plant diversity (*F*_1,64_ = 4.04, *P* = 0.048, Fig.3). Carnivorous nematodes of the genera *Nygolaimus*, *Paraxonchium,* and *Sectonema* were not found in the bare plots (data not shown).

### Prey responses

**Figure 4:**
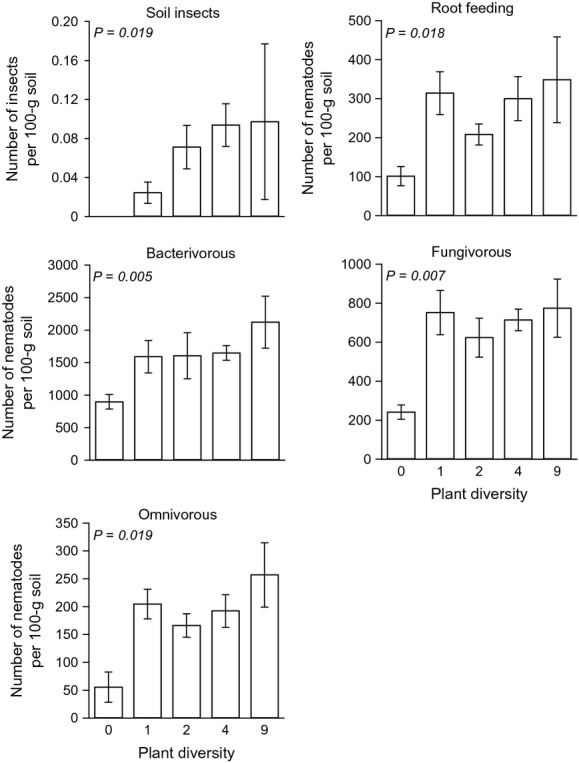
Effect of plant diversity on the abundance of prey: soil insects, and root-feeding, bacterivorous, fungivorous, and omnivorous nematodes. Means ± SE are shown. The number of nematodes and insects was calculated per 100 g dry weight soil. *P*-values are based on general linear model analyses where plant diversity was included as continuous variable.

**Figure 5:**
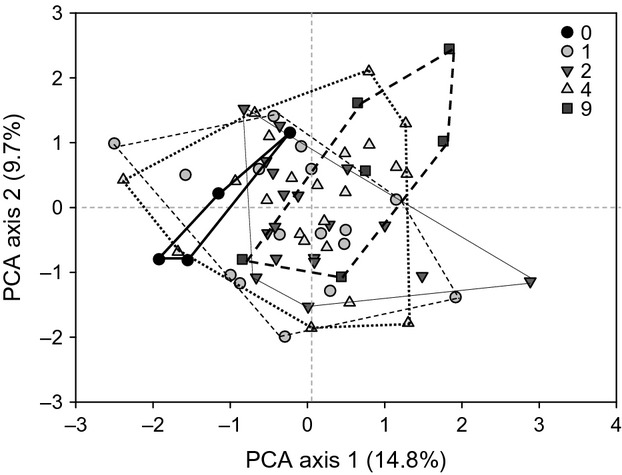
Ordination diagram of principal component analysis (PCA) of the prey nematode community in plots with 1, 2, 4, and 9 plant species and in bare plots (0). Percentages of total explained variation by PCA axes are given in parentheses.

The abundance of all noncarnivorous non-EP nematodes increased significantly with plant diversity, but the effect became nonsignificant when the bare plots were excluded from the analysis (Table1, Fig.4). The community composition of prey nematodes was also significantly related to plant diversity (RDA: *F* = 2.5, *P* = 0.002, Fig.5). There was a positive relationship between the proportion of legumes in a plant community and abundance of root-feeding, bacterivorous, and fungivorous nematodes. This was also true when bare plots were not included in the analysis (Table1). The proportion of grasses negatively affected fungivorous nematode abundance but stimulated the abundance of root-feeding nematodes (Table1). The abundance of root-feeding nematodes, however, decreased with increasing proportion of forbs (Table1). Abundances of root-feeding (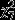 = 10.68, *P* = 0.15), bacterivorous (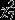 = 10.50, *P* = 0.16), fungivorous (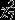 = 10.50, *P* = 0.16), and omnivorous (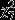 = 5.91, *P* = 0.55) nematodes did not differ between the monocultures. The majority of root-feeding insects that were recovered from the soil were white grubs. No insects were recovered from the soil collected from bare plots (Fig.4). There was a positive relationship between soil insect abundance and plant diversity when bare plots were included in the analysis (Table1, Fig.4). This relationship was marginally significant when bare plots were excluded from the model (*P* = 0.06). The density of soil insects was not affected by any of the three plant functional groups in the plant community (Table1).

### Plant community characteristics

There was no significant relationship between plant diversity and root biomass (Table1, Fig. S1A). However, root biomass positively correlated with the proportion of grasses in the community (Table1). Root biomass differed significantly between monocultures (*F*_7,8_ = 5.48, *P* = 0.014; Fig. S1B) and was highest in monocultures of *H. radicata* and *P. lanceolata*. Soil moisture content was not related to the diversity or identity of the plant community (all *P* > 0.05, Fig. S1, Appendix S3).

### Structural equation modeling

**Figure 6:**
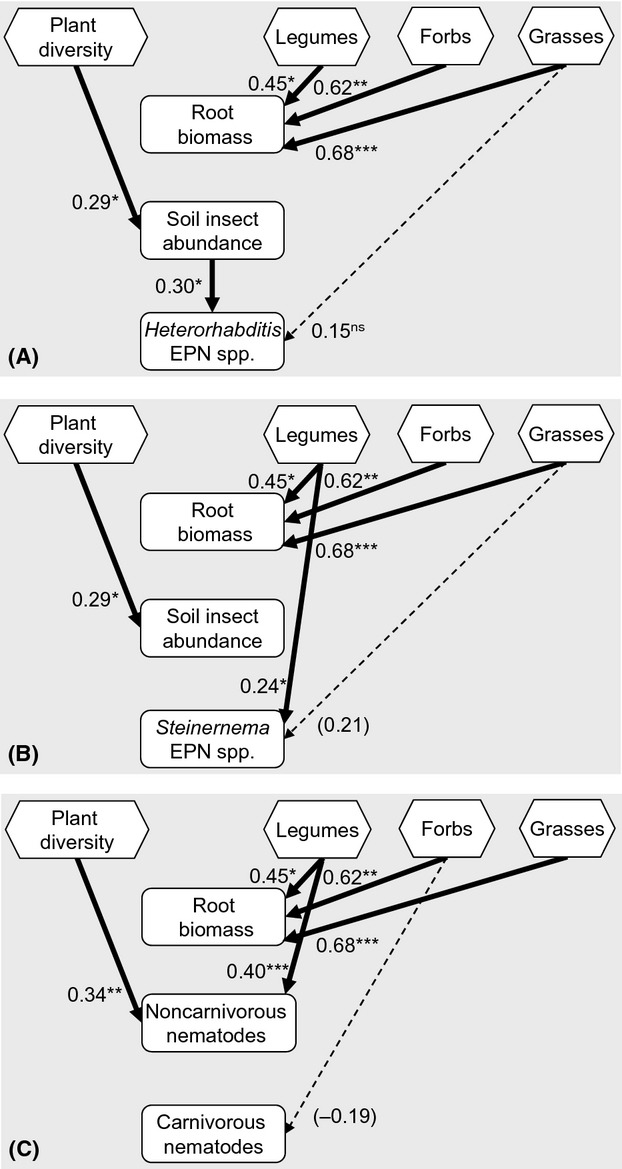
Final structural equation models for infectivity of (A) *Heterorhabditis* spp., (B) *Steinernema* spp., and (C) abundance of carnivorous non-EPNs. Values associated with arrows denote standardized path coefficients. Solid arrows indicate significant effects (at *P < 0.05, **P < 0.01, ***P < 0.001); dashed arrows with values in parentheses represent marginally nonsignificant effects (at *P* < 0.1); and dashed arrows with values (ns) represent nonsignificant effects kept in the model. The absence of arrows denotes nonsignificant effects that were removed from the model.

In the final SEM for *Heterorhabditis* spp. (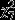 = 2.69, *P* = 0.98), 11.5% of the variation in percentage EPN infectivity could be explained by plant diversity and soil insect abundances (Fig.6A), which corresponds to hypothetical pathway A in Fig.1. For *Steinernema* spp. (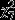 = 8.09, *P* = 0.53), there was a significant pathway between the percentage of EPN infectivity and the proportion of legumes in the community (Fig.6B). The pathway between plant diversity and soil insect abundance was also significant in this model (*P* = 0.014) and explained 8.6% of the variation in the soil insect abundance. The final SEM for carnivorous non-EPNs (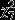 = 1.60, *P* = 0.66) did not reveal a significant pathway associated with their abundance (Fig.6C). There was a direct significant link between the abundance of noncarnivorous nematodes and plant diversity (*P* = 0.0014) and the proportion of legumes in the community (*P* > 0.001; Fig.6C) that explained 26.7% of the variation in their abundance. In all models, there was no significant pathway between predators and root biomass, thereby rejecting the hypothetical pathways B and C (Fig.1). Root biomass was significantly associated with the proportion of plant functional groups in the community (Fig.6A, B, C).

## Discussion

In our study, plant diversity positively affected the abundance of soil insects and nematode prey. However, the functioning (infectivity) of EPN spp. and the abundance of carnivorous non-EPNs were not directly affected by plant diversity. Interestingly, although there was no direct effect of plant diversity on the infectivity of EPN spp. in our study, the structural equation modeling revealed a significant indirect effect of plant diversity on *Heterorhabditis* infectivity via changes in the abundance of soil insects. These effects of plant diversity on *Heterorhabditis* EPNs are in line with pathway A (Fig.1) and the diversity-trophic structure hypothesis, which states that a greater number of resources support a greater number of consumers (Hutchinson 1959). Plant diversity, neither directly nor indirectly, affected the abundance of carnivorous non-EPNs and infectivity by *Steinernema*, suggesting that plant diversity effects might be genus or even species specific and that generalizations about diversity effects on soil organisms should be made with great caution.

The effect of plant identity was not consistent among and between the two genera of EPNs and the carnivorous nematodes. The abundance of carnivorous nematodes was not affected by the presence of particular functional groups although the abundance of their prey (root-feeding, bacterivorous, and fungivorous nematodes) was positively influenced by the proportion of legumes in the community. SEM also revealed a positive effect of legumes on the abundance of prey of the carnivorous nematodes. The positive effect of legumes might be explained by higher tissue nitrogen contents of plant roots or litter in presence of legumes that can lead to increased performance of root feeders and decomposers. Surprisingly, we did not observe an overall positive effect of legumes on the abundance of soil insects. This may be explained by the fact that root exudates of a large number of legumes contain isoflavonoids, which deter belowground insect larvae (Dakora 2003). It is important to note that the number of soil insects retrieved from the field plots in our study was low. *Steinernema* spp. infectivity was relatively high in the monocultures of the leguminous species *T. repens*, and according to SEM *Steinernema,* infectivity was positively affected by the presence of legumes. Increases in the abundance of predators in the soil can potentially lead to increased predation rates and as a result lower prey abundance (Siemann 1998; Preisser 2003). This suggests that potentially EPNs (in particular *Steinernema* species) could have reduced population densities of soil insects in legume plots. The infectivity of *Steinernema* spp. was also relatively high in the two monocultures of the grass species *F. rubra*. This might be explained by large amounts of fine roots produced by grass species altering soil structure and microclimate (but not soil moisture content) that potentially serves as beneficial habitat for EPNs (Lawrence et al. 2006). In our study, we could not discriminate between functional group and species identity effects for grasses as only the monoculture of *F. rubra* was included. Interestingly, no infection of wax moth larvae by *Steinernema* occurred in the monocultures of *A. millefolium*, whereas other study have shown that *A. millefolium* has a positive effect on free-living nematodes (Viketoft et al. 2005). For *Heterorhabditis* spp. infectivity, we did not observe any significant effects of plant identity. As our results differ from those obtained in other studies (e.g., De Deyn et al. 2004a; Viketoft et al. 2005, 2009), it appears that site-specific differences such as pool of plant species, nematode species present, and the history of the site are important for soil predatory invertebrates.

The infection rates of wax moth larvae by *Heterorhabditis* spp. were higher than by *Steinernema* spp., but in general, the infection rates for both genera were low. Although EPNs are widely distributed in soils of all sorts of ecosystems, there is considerable variability in EPN distribution across seasons and habitats (Stuart and Gaugler 1994; Spiridonov et al. 2007). The low infectivity and inconsistent results for the two EPN genera in our study may be the result of differences in local densities and patchy distributions of EPN populations (e.g., Lawrence et al. 2006; Spiridonov et al. 2007). Alternatively, the different responses of EPNs could be due to local differences in abiotic conditions or prey availability in the field. Soil moisture is one of the most important abiotic parameters for EPN survival (Lawrence et al. 2006). In our study, there was no difference in the soil moisture content between different plots and we cannot attribute the variation in the EPN abundances to variation in soil moisture unless that operated at finer spatial and temporal scales than we could measure. The majority of insect prey found in our study was scarab beetle larvae that are feeding on plant roots and typical hosts of EPNs that are dispersed in deeper soil layers, such as *Heterorhabditis*. Therefore, the difference in host availability and life histories between the two EPN genera might explain differences in EPN responses in our study with *Heterorhabditis* responding more strongly to general insect host abundance than *Steinernema*.

In contrast to our hypothesis and in line with several other studies (e.g., Spehn et al. 2000; Gastine et al. 2003), root biomass was not affected by plant diversity at the time scale of our experiment, while aboveground biomass increased with increasing plant diversity (Kostenko O. and Bezemer T.M. unpubl. data). Correspondingly, the SEM also did not reveal a significant relationship between abundance of nematodes and soil insects and root biomass. In contrast, in aboveground communities, the effects of plant diversity on consumer diversity and abundance occur primarily via changes in plant biomass (Koricheva et al. 2000; Borer et al. 2012). One possible explanation for this discrepancy with the aboveground system is that soil organisms are generally not restricted by the quantity of primary resources and that belowground plant diversity effects are generally not mediated through root biomass (e.g., Bezemer et al. 2010). It is important to note that to maintain the initial plant species composition, the experimental communities were regularly hand-weeded. It is almost inevitable that part of the roots of the removed plants remained in the soil, even though the aboveground parts of these plants were removed entirely. This can also explain why there was some root biomass present in the bare plots in our experiment. Therefore, hand weeding could cause perturbations in belowground systems that obscure the pure effect of plant biomass in synthetic biodiversity experiments (Bezemer and van der Putten 2007; Roscher et al. 2013). This will be the case in both seed addition and plant removal experiments.

EPNs and predatory nematodes are broadly used in biological control programs to suppress pests of agricultural crops in soil and enhance crop yields (Peters 1996; Denno et al. 2008). In our study, where plant communities were manually manipulated, we could not estimate the effect of predation on plant survival and productivity, but our findings suggest that increasing plant diversity will have an indirect positive effect on EPN infectivity (in particular *Heterorhabditis* spp.). Studies in which the abundance of EPNs or other nematodes was manipulated experimentally have demonstrated that increased levels of predation can have a strong positive impact on plant survival, productivity, and diversity (van der Putten and van der Stoel 1998; Preisser 2003; Khan and Kim 2007). It should also be emphasized that carnivorous non-EPN and EPNs are only a part of the predaceous soil fauna. Other important groups of soil predators not estimated in our study (e.g., microarthropods, protists) can also be affected directly or indirectly by plant diversity and identity. Ultimately, understanding the relationships between plant diversity, plant community composition, and natural populations of predatory organisms in the soil may provide new insights in the functioning of soil communities and their use as biological control agents in managed and natural systems.

In conclusion, our study shows that abundance of (non-EP) carnivorous nematodes is not influenced by the diversity or identity of the community, although their prey is affected by both characteristics of the plant community. However, increasing plant species diversity enhances the level of predation by *Heterorhabditis* EPNs in the soil but only indirectly by affecting the abundance of their prey. In contrast, the level of predation by *Steinernema* EPNs is not affected by an increase in prey abundance but is directly influenced by the composition/identity of the plant community. Thus, the responses of belowground organisms to manipulation in plant diversity and identity can be specific and may differ even between organisms that belong to different species but the same feeding guild, such as EPNs of the genera *Steinernema* and *Heterorhabditis*.

## Supporting Information

**Appendix S1.** Soil nematode extraction. **Appendix S2.** Structural equation modelling procedure. **Appendix S3.** Plant community characteristics. **Table S1.** Number of nematodes per 100 g dry soil (mean ± SE between plots), feeding type and taxa composition according to Yeates et al. (1993), Bongers (1994) and Andrássy (2005). **Table S2.** Standardized coefficients of the reciprocal pathways between the fixed factors in SEM models. **Figure S1.** Community root biomass in the plots with different plant diversity (A) and in different monocultures (B); and soil moisture content in the plots with different plant diversity (C). Means ± SE are shown.
