# Supplementary material for: Plant diversity and identity effects on predatory nematodes and their prey
Source: Ecol Evol. 2015 Jan 23;5(4):836–47. doi: 10.1002/ece3.1337 (PMC4338967; doi:10.1002/ece3.1337)
Supplement: Supplementary file 1 [file ece30005-0836-sd1.docx]

**Appendix S1**

*Soil nematode extraction*

The nematodes were extracted from fresh soil using the Oostenbrik elutriator technique (Oostenbrink 1960). For each sample this method comprises addition of 100 ml of soil to the water in the elutriator and subsequent collection of the soil suspensions with nematodes on a series of one 75 µm and three 45 µm mesh-sized sieves. The material on the sieves was gently and with little water put on a double cotton filter on a sieve which was then placed in a dish with 100 ml of tap water. The nematodes were allowed to migrate through the cotton filter into the water for 24 h at room temperature. The nematodes were collected in 100 ml jars and concentrated into 10 ml vials and subsequently into 2 ml of water by letting the nematodes settle to the bottom of the jars/vial and careful removal of the top layer of water. The concentrated nematode samples were then fixated by adding 4 ml of hot and 4 ml of cold 4% formalin. The total number of nematodes were then determined for each soil sample using a reversed-light microscope.

**Reference**

Oostenbrink, M. (1960) Estimating nematode populations by some selected methods. Nematology **6**: 85-102.Table S1. Number of nematodes per 100 g dry soil (mean ± SE between plots), feeding type and taxa composition according to Yeates et al. (1993), Bongers (1994) and Andrássy (2005). The soil cores (15 cm depth and 5 cm diameter) were collected from the inner 2.5 × 2.5 m square of each experimental plot (3 × 3 m) in a regular 0.5 × 0.5 m grid.

| Order | Family | Genus | Abundance ± SE | | | | |
| --- | --- | --- | --- | --- | --- | --- | --- |
| **Root-feeding** |  |  |  |  |  | |  |
| Diphtherophorida | Trichodoridae | *Trichodorus* | 7.0 | ± | 1.9 | |  |
| Dorylaimida | Nordiidae | *Pungentus* | 23.4 | ± | 3.2 | |  |
| Tylenchida | Anguinidae | *Ditylenchus* | 7.5 | ± | 1.4 | |  |
| Tylenchida | Hoplolaimidae | *Helicotylenchus* | 2.7 | ± | 1.6 | |  |
| Tylenchida | Meloidogynidae | *Meloidogyne* | 41.4 | ± | 6.8 | |  |
| Tylenchida | Paratylenchidae | *Paratylenchus* | 106.9 | ± | 21.7 | |  |
| Tylenchida | Pratylenchidae | *Pratylenchus* | 35.0 | ± | 8.8 | |  |
| Tylenchida | Tylenchidae | *Filenchus* | 15.3 | ± | 2.7 | |  |
| Tylenchida | Tylenchidae | *Malenchus* | 0.2 | ± | 0.2 | |  |
| Tylenchina | Telotylenchidae^a^ | *Tylenchorhynchus* | 17.5 | ± | 4.8 | |  |
| Tylenchida | Tylenchidae | Other Tylenchidae | 13.8 | ± | 3.4 | |  |
|  |  |  |  |  | |  | |
| **Bacterivorous** |  |  |  |  | |  | |
| Alaimida | Alaimidae | *Alaimus* | 13.9 | ± | | 2.1 | |
| Alaimida | Amphidelidae^e^ | *Paramphidelus* | 0.7 | ± | | 0.5 | |
| Araeolaimida | Bastianiidae | *Bastiania* | 2.1 | ± | | 1.2 | |
| Araeolaimida | Cylindrolaimidae | *Cylindrolaimus* | 5.0 | ± | | 1.7 | |
| Araeolaimida | Metateratocephalidae^d^ | *Metateratocephalus* | 0.3 | ± | | 0.3 | |
| Araeolaimida | Plectidae | *Anaplectus* | 63.7 | ± | | 10.0 | |
| Araeolaimida | Plectidae | *Plectus* | 187.3 | ± | | 22.9 | |
| Araeolaimida | Plectidae | *Tylocephalus* | 52.8 | ± | | 12.2 | |
| Araeolaimida | Plectidae | *Wilsonema* | 28.8 | ± | | 3.4 | |
| Enoplida | Prismatolaimidae | *Prismatolaimus* | 56.0 | ± | | 6.6 | |
| Monhysterida | Monhysteridae | *Eumonhystera* | 5.4 | ± | | 1.5 | |
| Monhysterida | Monhysteridae | *Monhystera* | 0.9 | ± | | 0.6 | |
| Rhabditida | Cephalobidae | *Acrobeles* | 444.1 | ± | | 27.7 | |
| Rhabditida | Cephalobidae | *Acrobeloides* | 332.5 | ± | | 24.8 | |
| Rhabditida | Cephalobidae | *Acrolobus* | 2.4 | ± | | 1.0 | |
| Rhabditida | Bunonematidae | *Bunonema* | 3.4 | ± | | 1.5 | |
| Rhabditida | Cephalobidae | *Cervidellus* | 19.9 | ± | | 3.9 | |
| Rhabditida | Cephalobidae | *Chiloplacus* | 6.1 | ± | | 1.7 | |
| Rhabditida | Cephalobidae^b^ | *Eucephalobus* | 129.1 | ± | | 22.2 | |
| Rhabditida | Mesorhabditidae^c^ | *Mesorhabditis* | 1.9 | ± | | 1.1 | |
| Rhabditida | Panagrolaimidae | *Panagrolaimus* | 94.5 | ± | | 18.5 | |
| Rhabditida | Rhabditidae |  | 162.4 | ± | | 35.5 | |
| Rhabditida | Teratocephalidae | *Teratocephalus* | 7.0 | ± | | 3.4 | |
|  |  |  |  |  | |  | |
| **Fungivorous** |  |  |  |  | |  | |
| Aphelenchida | Aphelenchidae | *Aphelenchus* | 385.4 | ± | | 30.1 | |
| Aphelenchida | Aphelenchoididae | *Aphelenchoides* | 259.7 | ± | | 29.1 | |
| Diphtherophorida | Diphtherophoridae | *Diphterophora* | 24.4 | ± | | 3.2 | |
| Dorylaimida | Tylencholaimidae^f^ | *Tylencholaimus* | 6.0 | ± | | 1.6 | |
|  |  |  |  |  | |  | |
| **Omnivorous** |  |  |  |  | |  | |
| Dorylaimida | Dorylaimidae^i^ | *Mesodorylaimus* | 13.5 | ± | | 3.8 | |
| Dorylaimida | Qudsianematidae | *Crassolabium*^j^ | 118.2 | ± | | 9.8 | |
| Dorylaimida | Qudsianematidae | *Dorydorella* | 11.0 | ± | | 2.5 | |
| Dorylaimida | Qudsianematidae^h^ | *Ecumenicus* | 22.9 | ± | | 4.2 | |
| Dorylaimida | Qudsianematidae | *Epidorylaimus* | 1.4 | ± | | 0.8 | |
| Dorylaimida | Qudsianematidae | *Eudorylaimus* | 2.5 | ± | | 0.9 | |
| Dorylaimida | Qudsianematidae | *Microdorylaimus* | 16.3 | ± | | 3.7 | |
|  |  |  |  |  | |  | |
| **Carnivorous** |  |  |  |  | |  | |
| Dorylaimida | Aporcelaimidae | *Aporcelaimus* | 59.0 | ± | | 4.6 | |
| Dorylaimida | Mydonomidae^g^ | *Dorylaimoides* | 17.9 | ± | | 4.8 | |
| Dorylaimida | Nygolaimidae | *Nygolaimus* | 0.3 | ± | | 0.3 | |
| Dorylaimida | Nygolaimidae | *Sectonema* | 0.3 | ± | | 0.3 | |
| Dorylaimida | Paraxonchiidae^k^ | *Paraxonchium* | 5.2 | ± | | 1.6 | |
| Mononchida | Mononchidae |  | 141.9 | ± | | 11.9 | |
| Rhabditida | Neodiplogastridae |  | 8.2 | ± | | 2.1 | |
| According to Bongers (1994): ^a^Dolichodoridae; ^b^Diplopeltidae; ^c^Rhabditidae; ^d^Teratocephalidae; ^e^Alaimidae; ^f,g^Leptonchidae; ^h,i^Thornenematidae; ^j^*Thonus*; ^k^Aporcelaimidae. | | | | | | | |

**References**

Andrássy, I. (2005) *Free-living nematodes of Hungary: Nematoda errantia.* Budapest: Hungarian Natural History Museum: Systematic Zoology Research Group of the Hungarian Academy of Sciences, Budapest, Hungary.

Bongers, T. (1994) *De Nematoden van Nederland.* Pirola, Schoorl, Utrecht, The Netherlands.

Yeates, G.W., Bongers, T., de Goede, R.G.M., Freckman, D.W. & Georgieva, S.S. (1993) Feeding-habits in soil nematode families and genera - an outline for soil ecologists. *Journal of Nematology,* **25,** 315-331.

**Appendix S2**

*Structural equation modelling procedure*

All variables used in the SEM were observed variables. The reciprocal effects of soil insects or root-feeding nematodes on plant root biomass and higher trophic level organisms on their prey were excluded from the initial model because plant diversity was experimentally manipulated in our study.

Table S2. Standardized coefficients of the reciprocal pathways between the fixed factors in SEM models.

|  | Plant diversity | Legumes | Forbs | Grasses |
| --- | --- | --- | --- | --- |
| Plant diversity | 1 | -0.03 | 0.20 | 0.15 |
| Legumes | -0.03 | 1 | -0.52 | -0.20 |
| Forbs | 0.20 | -0.52 | 1 | -0.56 |
| Grasses | 0.15 | -0.20 | -0.56 | 1 |

**Appendix S3**

Fig. S1. Community root biomass in the plots with different plant diversity (A) and in different monocultures (B); and soil moisture content in the plots with different plant diversity (C). Means ± SE are shown. Different letters denote significant differences between monocultures (*P* < 0.05) based on a Tukey HSD test.
